# From genome guardian to immune modulator: the dual faces of DNA-PKcs

**DOI:** 10.1042/BSR20260587

**Published:** 2026-11-11

**Authors:** Qiang Li, Pei-Wen Zhan, Xian Hong, Ying Qu, Tao Wang

**Affiliations:** 1 Laboratory of Protein Structure and Function, Institute of Medicine and Pharmacy, Qiqihar Medical University, Qiqihar, Heilongjiang 161006, China; 2 School of Medical Technology, Qiqihar Medical University, Qiqihar, Heilongjiang 161006, China; 3 Laboratory of Experimental Diagnosis, the Second Affiliated Hospital of Qiqihar Medical University, Qiqihar, Heilongjiang 161006, China

**Keywords:** cGAS-STING, diseases, DNA Repair, DNA-PKcs, inhibitors, innate immunity

## Abstract

The DNA-dependent protein kinase catalytic subunit (DNA-PKcs) has long been recognized as a genome guardian, serving as a master regulator of non-homologous end joining (NHEJ), safeguarding genomic integrity and enabling adaptive immunity through V(D)J recombination. Beyond this nuclear function, DNA-PKcs is now increasingly appreciated as a context-dependent immune modulator, acting at the interface between the DNA damage response and inflammatory signaling. This review synthesizes recent advances that redefine DNA-PKcs as a versatile “molecular integrator.” We delineate its structural dynamics and multifaceted regulation, and dissect its dual function in the cytoplasm, where it can either potentiate or suppress the cyclic GMP–AMP synthase and stimulator of interferon genes (cGAS–STING) pathway and even initiate STING-independent interferon responses. We further explore how dysregulation of DNA-PKcs underpins a spectrum of pathologies, from immunodeficiency and autoimmunity to cancer and neurodegeneration. Finally, we discuss the translational promise of DNA-PKcs inhibitors, which not only act as classical chemo- and radio-sensitizers but also remodel the tumor microenvironment by activating antitumor immunity. Decoding the context-dependent logic behind these dual faces is therefore paramount for developing novel therapeutics that leverage DNA-PKcs across oncology, autoimmunity, and neurodegeneration.

## Introduction

The catalytic subunit of the DNA-dependent protein kinase (DNA-PKcs) is a core component of the DNA-PK complex, which serves as a molecular machine for initiating repair of double-strand breaks (DSBs), the most lethal form of DNA damage, in eukaryotic cells. As the central driver of non-homologous end joining (NHEJ), the predominant DSB repair pathway, DNA-PK maintains genomic integrity by rapidly recognizing DSBs and coordinating end processing and ligation [1]. Its function is crucial not only for fundamental cellular viability but also for adaptive immunity. By mediating coding end ligation during V(D)J recombination, DNA-PK provides the molecular basis for generating the diverse T- and B-cell receptor repertoire, with its functional deficiency directly leading to severe combined immunodeficiency (SCID) [2,3].

Recent advances have positioned the DNA-PK complex as a critical bridge connecting the DNA damage response (DDR) and the innate immune system, revealing a functional paradigm that extends far beyond its canonical role in repair. The aberrant presence of DNA in the cytoplasm, a hallmark of genomic instability and a consequence of factors such as infection or cellular dysfunction, serves as a critical danger signal that potently activates the innate immune cyclic GMP–AMP synthase and stimulator of interferon genes (cGAS–STING) axis [4–6]. Intriguingly, DNA-PK, the nuclear “DNA repair specialist,” has been found to translocate into the cytoplasm and directly participate in regulating this immune pathway [7]. However, the regulatory role of DNA-PK in this immune pathway remains incompletely defined and controversial. Some studies indicate that DNA-PK suppresses immune responses by phosphorylating cGAS or recruiting Poly(ADP-ribose) polymerase 1 (PARP1), potentially preventing autoimmune damage [8,9]; whereas other evidence demonstrates that it enhances cGAS activity or directly phosphorylates interferon regulatory factor 3 (IRF3), thereby potentiating interferon production and antiviral defense [7,10,11]. This functional dichotomy, coupled with potential species-specific differences (such as the existence of a STING-independent pathway in human cells), underscores the complexity of DNA-PK’s role in immune homeostasis.

Given the pivotal position of DNA-PK as both a “guardian of the genome” and an “immune modulator,” its dysfunction is implicated in a spectrum of diseases, ranging from immunological disorders like SCID and systemic lupus erythematosus (SLE) to cancer and neurodegenerative diseases [12–16]. Concurrently, DNA-PKcs inhibitors, which disrupt DSB repair in tumor cells, have emerged as promising agents for sensitizing cancers to radiotherapy, chemotherapy, and PARP inhibitors.

In this review, we first summarize the structural organization, activation mechanisms, and post-translational regulation of DNA-PKcs, with emphasis on how these features support its canonical function in NHEJ. We then discuss the central role of DNA-PKcs in DSB repair and V(D)J recombination, highlighting its function as a guardian of genome integrity and adaptive immune development. Next, we examine emerging evidence that DNA-PKcs also acts as a context-dependent modulator of innate immune signaling, particularly through its positive and negative regulation of the cGAS–STING pathway and its ability to support STING-independent interferon responses. We further review how dysregulated DNA-PKcs activity contributes to immunodeficiency, autoimmunity, cancer, and neurodegeneration. Finally, we discuss current strategies for therapeutic targeting of DNA-PKcs, including its use as a chemo- and radiosensitizing target and its potential to reshape antitumor immunity. Together, this review aims to provide an integrated framework for understanding the dual faces of DNA-PKcs at the interface of genome maintenance and immune regulation.

## Structural insights into DNA-PKcs activation and regulation

### Assembly of DNA-PK complex

The DNA-PK is a pivotal complex in the DNA damage response, belonging to the phosphatidylinositol 3-kinase-related kinase (PIKK) family. Its holoenzyme consists of a large catalytic subunit, DNA-PKcs (encoded by the *PRKDC* gene), and the Ku heterodimer composed of Ku70 and Ku80. DNA-PKcs is not only the largest serine/threonine kinase in the PIKK family but also one of the most abundantly expressed members in human cells [17,18]. The Ku heterodimer serves as the initial DNA-end sensor, exhibiting high affinity for DSB termini [19]. Upon binding to DNA ends, Ku undergoes a conformational change that facilitates the recruitment of DNA-PKcs, leading to the assembly of the active DNA-PK holoenzyme at the damage site. Ku not only positions DNA-PKcs spatially but also allosterically activates its kinase activity [20,21], marking a critical initiation event in the DDR.

### Domain architecture of DNA-PKcs

Structurally, DNA-PKcs is organized into three major units: a large N-terminal region, a middle circular cradle, and a C-terminal head [22,23] (Figure 1). The N-terminal region (residues 1–892) consists of 38 α-helices organized into four supersecondary structures. This region contributes to DNA binding and exhibits significant conformational flexibility, which is thought to facilitate allosteric kinase regulation and the eventual release of repaired DNA [23–25]. The middle circular cradle (residues 893–2801) comprises 86 α-helices forming five supersecondary structures. Together with the N-terminal unit, this cradle adopts a ring-like structure that creates a base through which Ku70/80 may present DNA ends for repair, and it also contains binding sites for Ku80. Both the N-terminal region and the middle circular cradle contain Huntingtin, Elongation factor 3, Protein phosphatase 2A, and TOR1 (HEAT) repeats. These repeats form a flexible, continuous scaffold that is critical for mediating protein–protein interactions and give rise to supersecondary structures with continuous hydrophobic cores [23,26]. The C-terminal head (residues 2802–4128) encompasses the FRAP–ATM–TRRAP (FAT) region (residues 2802–3564), the FKBP12-rapamycin-binding (FRB) domain (residues 3582–3675), the kinase domain (residues 3676–4100), and the FAT C-terminal motif (FATC) domain (residues 4101–4128). While the kinase domain shares structural homology with other phosphatidylinositol 3-kinase (PI3K) family members, it is distinguished by unique insertions, such as α-hairpins 1–3, which restrict access to the active site [23]. A key regulatory event involves the FRB domain, which undergoes an outward movement upon Ku binding, a regulatory mechanism analogous to that observed in mTOR (mechanistic target of rapamycin kinase) [27]. Collectively, the FAT, FRB, and FATC regions stabilize the conformational changes of the catalytic center and regulate kinase activity [22,23,26].

**Figure 1:**
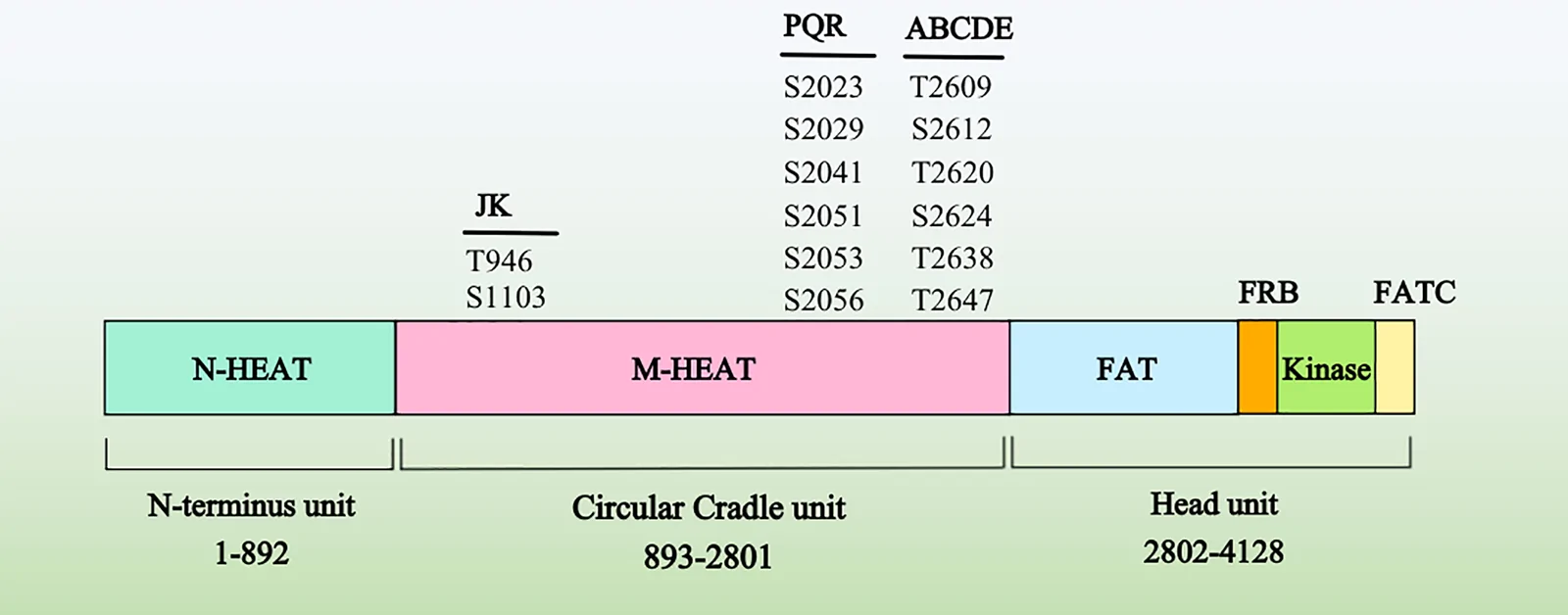
The functional domains and phosphorylation sites of DNA-PKcs Schematic representation of the three major structural regions of DNA-PKcs: the N-terminal unit, the middle circular cradle formed by the M-HEAT repeats, and the C-terminal head containing FAT, FRB, kinase, and FATC. The positions of the JK, PQR, and ABCDE autophosphorylation clusters within the M-HEAT repeat region are highlighted. The ABCDE and PQR clusters are key regulators of NHEJ progression, whereas the JK cluster modulates DSB repair-pathway choice toward HR without altering DNA-PKcs kinase activity.

### Post-translational modifications of DNA-PKcs

The activity and function of DNA-PKcs are finely tuned by phosphorylation and other post-translational modifications (PTMs). Within the middle circular cradle unit, two major phosphorylation clusters (ABCDE and PQR) play central roles in regulating DNA-PKcs dynamics (Figure 1). The ABCDE cluster (including T2609, S2612, T2620, S2624, T2638, and T2647) is critical for complex disassembly and repair progression [28]. Phosphorylation at T2609, a well-characterized radiation-induced site, regulates the access and endonuclease activity of Artemis, influencing both the hairpin-opening step of V(D)J recombination and the 5′ and 3′ overhang processing in NHEJ [29,30]. Beyond nuclear repair, T2609 phosphorylation also serves as a pivotal regulatory node in response to oxidative stress. Under such conditions, ataxia-telangiectasia mutated (ATM)-mediated T2609 phosphorylation triggers the dissociation of the mitochondrial DAV complex (comprising DNA-PKcs, ANT2, and VDAC2), thereby modulating mitochondrial membrane potential and oxidative phosphorylation [31]. Furthermore, in the presence of Ku and target DNA, phosphorylation of the T2647 site triggers a conformational change in DNA-PK, enhancing Artemis’ endonuclease activity at the junctions of single-stranded and double-stranded DNA, thereby ensuring efficient DNA double-strand break repair [32]. The PQR cluster (including S2023, S2029, S2041, S2051, S2053, and S2056) is vital for NHEJ efficiency. Autophosphorylation at S2056 induces a conformational change in DNA-PKcs, promoting its dissociation from DNA ends and facilitating subsequent ligation steps [29]. Additional autophosphorylation sites, such as T3950 and T4102, further modulate kinase activity and complex stability. Phosphorylation at T4102 by ATM enhances DNA-PKcs activity and its interaction with the Ku-DNA complex, promoting NHEJ and radioresistance [33,34]. In contrast with the ABCDE and PQR clusters that primarily regulate NHEJ progression, the JK cluster (T946, S1004), another autophosphorylation cluster in the circular cradle unit, acts as a molecular switch for DSB repair pathway choice. Phosphorylation of the JK cluster suppresses NHEJ and shifts DNA repair toward homologous recombination (HR), without affecting the intrinsic kinase activity of DNA-PKcs [35–37].

Beyond phosphorylation, DNA-PKcs is also regulated by acetylation, ubiquitination and PARylation. Acetylation at K3241 and K3260 impairs DSB repair, while deacetylation by SIRT2 enhances Ku interaction and DNA-PK activity [38,39]. Several E3 ligases, including Ring Finger protein 144A (RNF144A) and Cullin-RING ubiquitin Ligase 4A complex with substrate receptor DTL (CRL4A^DTL^), target DNA-PKcs for ubiquitination and degradation in the cytoplasm and nucleus, respectively [40,41]. HECT, UBA, and WWE domain containing E3 ubiquitin protein ligase 1 (HUWE1)-mediated neddylation of DNA-PKcs promotes S2056 autophosphorylation and NHEJ efficiency [42]. Additionally, PARP1-mediated PARylation of DNA-PKcs fine-tunes its phosphorylation status, further integrating metabolic and damage signals into the repair process [43].

### Activation and regulatory network of DNA-PKcs

DNA-PKcs activation is initiated by Ku binding to DSB ends, which recruits DNA-PKcs and induces a conformational change that activates its kinase activity [44–46]. This process is further fine-tuned by a multi-layered network of post-translational modifications and regulatory factors.

On the one hand, several proteins positively regulate DNA-PKcs function. For instance, the histone acetyltransferase TIP60, epidermal growth factor receptor (EGFR), and protein phosphatase 1 (PP1) interact with DNA-PKcs and promote DNA-PKcs activation [47–49]. The deacetylase Sirtuin 2 (SIRT2) interacts with and deacetylates DNA-PKcs, which strengthens the DNA-PKcs/Ku interaction and promotes downstream NHEJ processes [38]. Additionally, the methyltransferase SET and MYND domain-containing protein 2 (SMYD2) promotes NHEJ complex assembly and efficiency by methylating Ku70 [50]. Moreover, the deubiquitinase Ubiquitin-Specific Protease 7 (USP7) stabilizes DNA-PKcs by removing its ubiquitin modifications, a process that is potentiated by cGAS [51]. Our work has further identified the Wiskott–Aldrich Syndrome Protein and SCAR Homolog (WASH) complex as a critical positive regulator of DNA-PK. We demonstrated that WASH interacts with the DNA-PK complex, promoting DNA-PKcs activation, catalyzing H2AX and KAP1 phosphorylation, and facilitating the recruitment of downstream repair factors to enhance NHEJ efficiency [52]. We also elucidated the molecular basis of this regulation by showing that the WASH-associated protein FAM21 interacts with Ku proteins via its C-terminus, thereby recruiting the WASH complex to DSB sites and solidifying the WASH/FAM21–DNA-PK axis [53].

On the other hand, multiple factors negatively regulate DNA-PKcs activity. The non-coding RNA scaRNA2 inhibits NHEJ by interfering with the DNA–PKcs–Ku interaction [54]. The E3 ubiquitin ligase RNF144A promotes the ubiquitination and subsequent degradation of DNA-PKcs [40]. Notably, we recently uncovered that VPS35 exerts a negative regulatory role on the DNA-PK complex by interacting with and sequestering Ku, which impedes its recruitment to DSBs and consequently attenuates DNA-PKcs activation and overall NHEJ efficiency [55]. In summary, the activation and function of DNA-PK are governed by a sophisticated regulatory system involving structural rearrangement, phosphorylation cascades, and a diverse array of post-translational modifications, collectively ensuring precise control of DSB repair.

## The central role of DNA-PKcs in NHEJ

Among the various types of DNA damage, DSBs represent the most cytotoxic lesions, with the potential to cause genomic instability, chromosomal translocations, and cell death if left unrepaired [56,57]. To counter this threat, eukaryotic cells primarily utilize the NHEJ pathway, a rapid, template-independent repair mechanism that functions throughout the cell cycle [58]. While error-prone, NHEJ is indispensable for maintaining genomic integrity and, as detailed in this section, the DNA-PKcs sits at its functional core.

### The stepwise NHEJ mechanism

The NHEJ repair cascade is initiated by the Ku70/Ku80 heterodimer, which possesses a pre-formed ring structure that allows it to slide onto DSB ends with high affinity and specificity [59] (Figure 2). This binding serves a dual purpose: it protects the DNA termini from excessive resection and acts as an essential recruitment platform for subsequent factors. The pivotal next step is the recruitment of the catalytic subunit DNA-PKcs by the Ku-DNA complex, forming the complete DNA-PK holoenzyme. The assembly of this complex at the break site triggers a DNA-induced conformational change that activates the serine/threonine kinase activity of DNA-PKcs.

**Figure 2:**
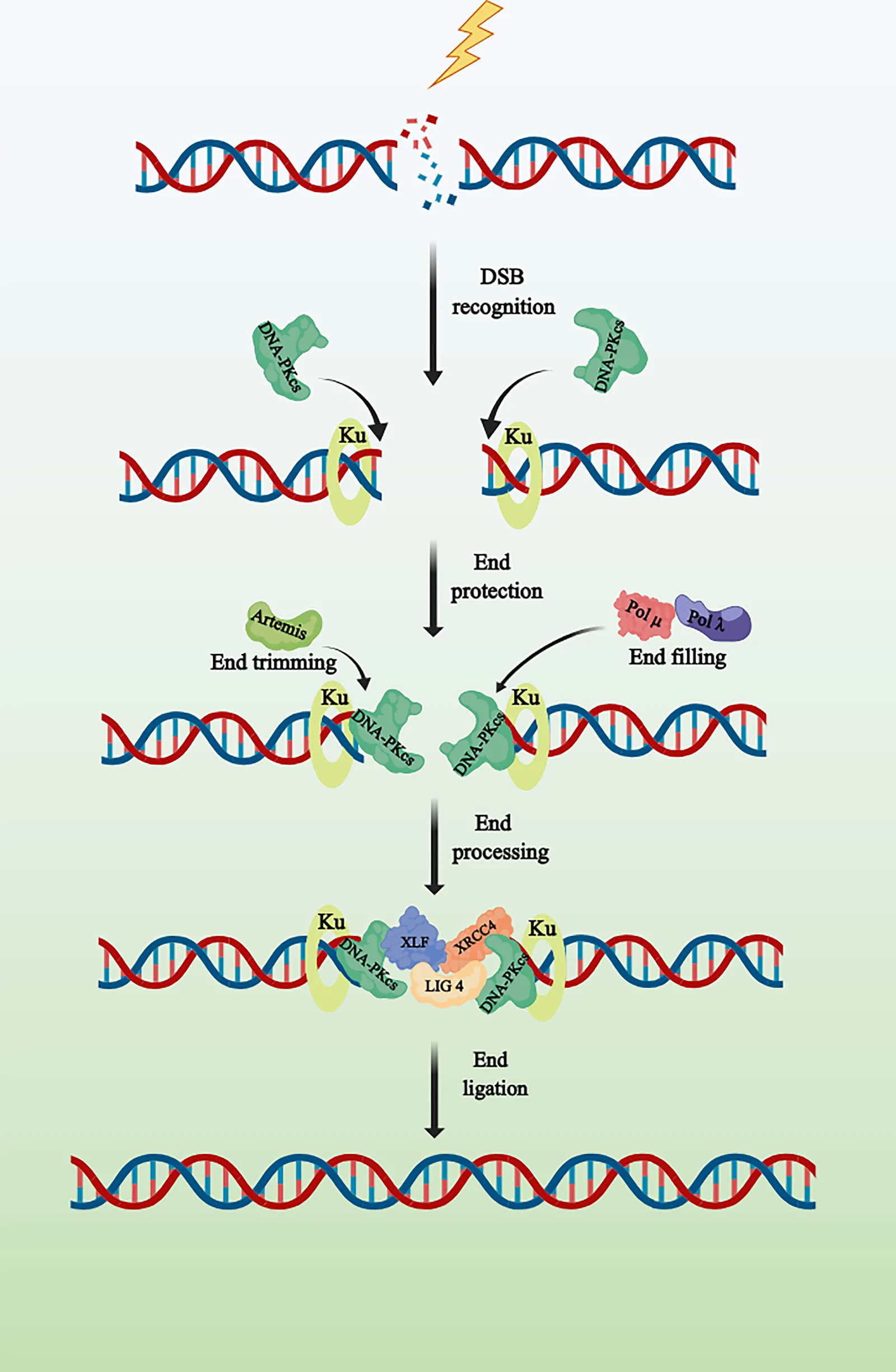
The DSB repair of NHEJ DSBs are first recognized by the Ku70/Ku80 heterodimer, which loads onto broken DNA termini and recruits DNA-PKcs to assemble the DNA-PK holoenzyme. Upon activation, DNA-PKcs coordinates sequential end-processing events. Artemis nuclease conducts end-trimming of overhanging or complex DNA ends, while Pol μ and Pol λ carry out end-filling to generate compatible ligatable termini. Finally, the processed DNA ends are subsequently joined by the XRCC4-DNA Ligase IV complex stabilized by XLF, completing DSB repair.

Once activated, DNA-PKcs orchestrates the subsequent steps of NHEJ. It phosphorylates itself at key residues, such as Ser2056 within the PQR cluster, which induces a substantial conformational change that promotes the dissociation of the DNA-PK complex from the DNA ends [29,60]. This “clearing the deck” step is crucial, as it makes the processed ends accessible for the final ligation machinery. DNA-PKcs also phosphorylates downstream effectors; for instance, phosphorylation of XRCC4 enhances its interaction with DNA Ligase IV, promoting the stable recruitment of this critical ligase to the damage site [61].

Many DSB ends are not immediately ligatable and require processing by nucleases and polymerases. DNA-PKcs plays a central role in activating these enzymes. A key target is the Artemis nuclease, which is phosphorylated and activated by DNA-PKcs. Artemis mediates end-trimming of overhanging DNA termini and is essential for resolving complex DSB ends, such as the hairpin structures generated during V(D)J recombination [62,63]. Subsequently, as the major Pol X-family polymerases in NHEJ, Pol μ and Pol λ mediate end-filling through both template-dependent and template-independent mechanisms [64,65]. These polymerases remodel broken DNA ends to yield compatible termini, preparing break ends for subsequent ligation. Finally, the XRCC4-DNA Ligase IV complex, stabilized by XLF, performs the ligation step, resealing the DNA backbone to complete the repair process [66].

### DNA-PK directs repair pathway choice

Beyond its direct role in NHEJ execution, DNA-PK is a critical arbitrator in the cellular decision between NHEJ and the high-fidelity homologous recombination (HR) pathway. A key regulatory point is the initiation of DNA end resection, which generates 3′ single-stranded DNA overhangs essential for HR and is inherently antagonistic to NHEJ. DNA-PK actively suppresses excessive resection in the G1 phase of the cell cycle, thereby promoting NHEJ. One established mechanism involves the DNA-PKcs-dependent up-regulation of RBX1, which enhances the neddylation of Cullin1 and subsequently targets the resection nuclease EXO1 for degradation, effectively limiting HR initiation in G1 [67].

As cells progress into S and G2 phases, where a sister chromatid template becomes available for high-fidelity repair, the balance shifts toward HR [68,69]. This transition is facilitated by the context-specific suppression of DNA-PK activity [35]. Multiple mechanisms contribute to this regulation. For instance, BRCA1 directly interacts with and inhibits DNA-PKcs autophosphorylation, thereby relieving the suppression on end resection and promoting HR factor loading at DSBs [70]. Additionally, SUMOylation of the acetyltransferase TIP60 by the E3 ligase PIAS4 disrupts its interaction with DNA-PKcs, reducing DNA-PKcs autophosphorylation at S2056 and further promoting the HR-friendly environment [71]. Thus, through tightly regulated activity and specific protein interactions, DNA-PK ensures the appropriate repair pathway is engaged in a cell cycle-dependent manner.

### DNA-PK in V(D)J recombination

The indispensable role of DNA-PK in NHEJ is vividly illustrated by its function in V(D)J recombination, the process that generates the diverse repertoire of T-cell and B-cell antigen receptors in the vertebrate adaptive immune system [72]. This process is initiated by the recombination-activating gene 1/recombination-activating gene 2 (RAG1/RAG2) complex, which introduces programmed DSBs between coding segments and recombination signal sequences, resulting in coding ends with closed hairpin structures [73].

The DNA-PK complex is rapidly recruited to these breaks. Ku binds the DNA ends, recruits DNA-PKcs, and the activated kinase then phosphorylates and activates the Artemis nuclease [74]. Artemis opens the hairpin structures, and after further processing by other nucleases and polymerases, the coding ends are ligated by the DNA Ligase IV complex [63,75]. The critical nature of DNA-PK in this process is demonstrated by the severe consequences of its dysfunction. Mutations in the gene encoding DNA-PKcs, *PRKDC*, abrogate coding joint formation, preventing the development of functional T and B lymphocytes and resulting in SCID [3,76]. This direct link between a DNA repair protein and immune competence underscores the physiological paramountcy of DNA-PK and the NHEJ pathway.

## DNA-PK in innate immunity

The innate immune system serves as the first line of defense against pathogenic invasion and cellular damage. Genomic instability and DNA damage can lead to the leakage of self-DNA into the cytoplasm. This misplaced DNA, acting as a damage-associated molecular pattern (DAMP), is sensed by pattern recognition receptors (PRRs) to trigger potent immune responses [4,77,78]. Among the cytosolic DNA sensors, the cGAS–STING pathway has emerged as the cornerstone of DNA-induced innate immunity. The cGAS binds to double-stranded DNA, undergoes liquid-phase condensation and dimerization, and catalyzes the synthesis of the second messenger 2′3′-cGAMP [79–81]. cGAMP then activates the adapter protein STING on the endoplasmic reticulum, leading to its translocation to the Golgi apparatus, where it recruits and activates TANK-binding kinase 1 (TBK1) [82–84]. TBK1 subsequently phosphorylates IRF3, prompting its dimerization, nuclear translocation, and the induction of type I interferons (IFNs) and other inflammatory cytokines [85] (Figure 3).

**Figure 3:**
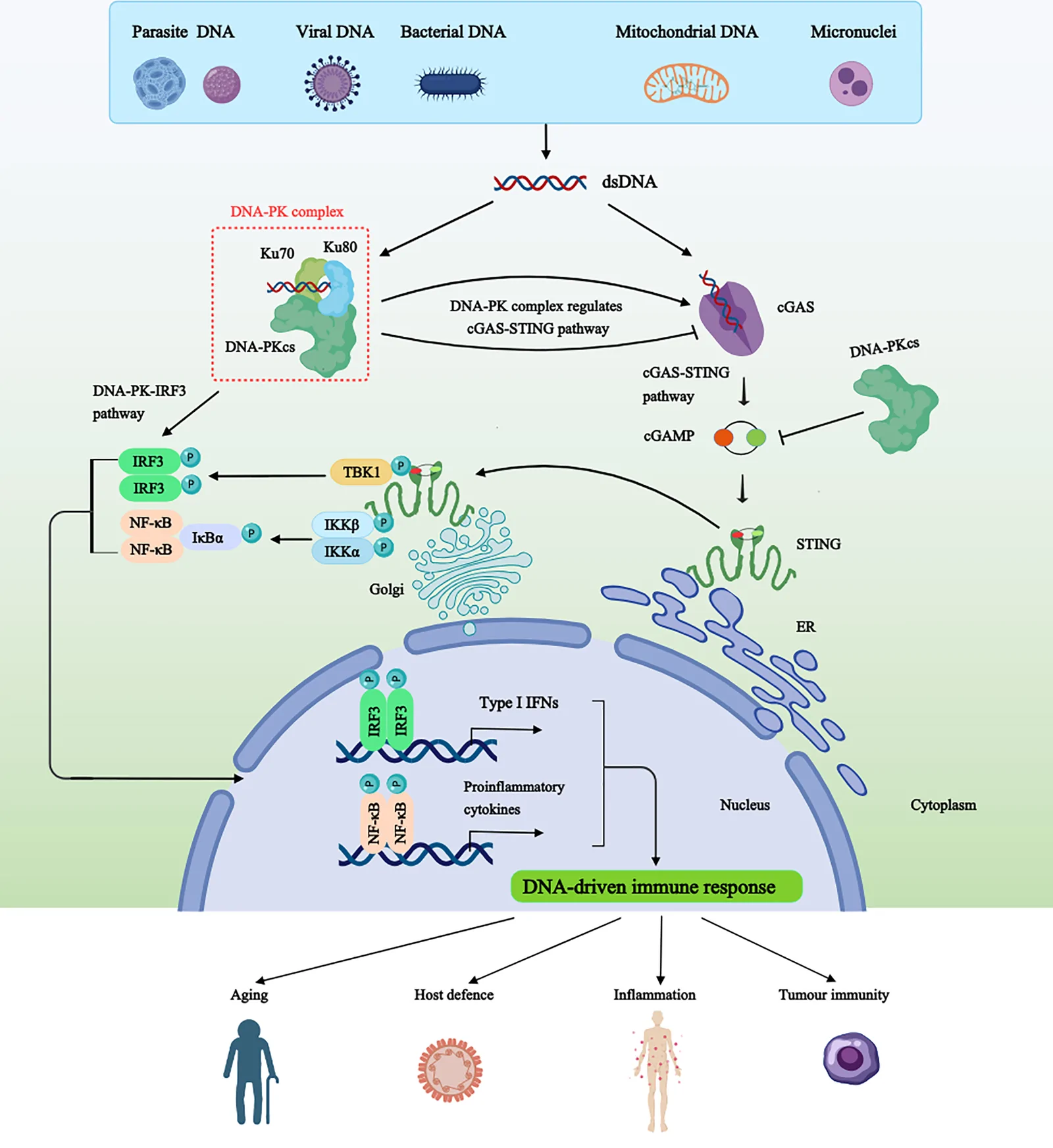
Context-dependent immunomodulation by DNA-PKcs in cytosolic DNA-sensing pathways Schematic diagram showing cytosolic dsDNA from diverse sources, including parasitic, viral, bacterial, mitochondrial DNA and micronuclei, which initiates innate immune signalling. In the canonical cGAS–STING cascade, dsDNA activates cGAS to generate 2′3′-cGAMP; cGAMP binds and activates STING at the endoplasmic reticulum, triggering STING translocation to the Golgi apparatus and subsequent TBK1-dependent phosphorylation of IRF3 and IKK-mediated NF-κB activation. Activated IRF3 and NF-κB translocate into the nucleus to induce expression of type I interferons and proinflammatory cytokines. The DNA-PK complex (Ku70/Ku80/DNA-PKcs) acts as a context-dependent rheostat to fine-tune cGAS-STING signaling. DNA-PKcs regulates cGAS activation through post-translational modification, thereby either promoting or inhibiting cGAS catalytic activity. In addition, DNA-PKcs directly binds 2′3′-cGAMP and thereby suppresses downstream STING activation. Separately, in a STING-independent pathway reported in certain human cells, DNA-PKcs can bind cytosolic dsDNA and promote IRF3 phosphorylation, leading to type I interferon production. Collectively, these immune outputs regulate host defense, inflammation, tumor immunity, and aging.

Intriguingly, the DNA-PKcs, a well-established core component of the canonical non-homologous end joining (c-NHEJ) pathway in the nucleus, has been found to play a multifaceted and controversial role in regulating this critical immune signaling cascade.

### DNA-PK modulates cGAS–STING signaling

The role of DNA-PK in cytosolic DNA sensing is paradoxical, with compelling evidence supporting both inhibitory and stimulatory functions, likely dependent on cellular context, stimulus, and species.

Multiple lines of evidence position DNA-PK as a negative regulator of the pathway. Notable work in human THP-1 monocytic cells revealed that DNA-PKcs directly phosphorylates cGAS, markedly attenuating its enzymatic activity and leading to reduced 2′3′-cGAMP production and suppressed expression of immune genes such as IFNB1 and CXCL10 [8]. This mechanism is proposed to prevent excessive immune activation in response to self-DNA, offering a plausible explanation for the autoimmunity associated with specific *PRKDC* missense mutations. Further expanding this inhibitory axis, studies have shown that during viral infection, DNA-PKcs phosphorylates nuclear PARP1 at threonine 594, facilitating its translocation to the cytoplasm. There, PARP1 catalyzes the PARylation of cGAS, obstructing its DNA-binding capacity and thereby blunting the antiviral interferon response [9].

In contrast, several research reports based on human cell lines and mouse models support a positive regulatory role for the DNA-PK complex in innate immune signaling. The Ku70/Ku80 heterodimer has been shown to interact directly with cGAS, enhancing its DNA-binding affinity and promoting the formation of functionally active cGAS aggregates, which amplifies immune signaling [86]. Additionally, a specific activating mechanism has been identified wherein DNA-PKcs phosphorylates cGAS at serine 435, significantly boosting its catalytic efficiency in cGAMP production [11]. Beyond modulating cGAS, DNA-PKcs can also directly phosphorylate IRF3, promoting its dimerization and nuclear translocation, a modification essential for IRF3-dependent antiviral responses in models of Modified Vaccinia Ankara (MVA) and Herpes Simplex Virus-1 (HSV-1) infection [7,87]. Consistent with these findings, another study confirms that DNA-PKcs promotes DNA-induced activation of the STING/IRF3 axis and up-regulates the expression of innate immune genes [10].

Taken together, these seemingly opposing observations suggest that DNA-PKcs should not be viewed simply as either an activator or an inhibitor of cGAS–STING signaling, but rather as a context-dependent rheostat that shapes the amplitude and duration of DNA-induced innate immune responses. One possible reconciliation is that DNA-PKcs exerts temporally distinct functions during cytosolic DNA sensing. At the early phase of stimulation, DNA-PKcs may contribute to immune activation by sensing cytosolic dsDNA, facilitating cGAS activation, promoting cGAS aggregation or phosphorylation, and/or directly phosphorylating IRF3. These activities would help initiate or amplify antiviral signaling. In contrast, once cGAS has generated sufficient amounts of 2′3′-cGAMP and STING activation is underway, DNA-PKcs may switch to a signal-limiting role to prevent excessive or chronic inflammation. Supporting this model, a recent study showed that DNA-PKcs directly interacts with cyclic dinucleotides, including the endogenous second messenger 2′3′-cGAMP and the bacterial CDN 3′3′-cGAMP, through its kinase domain [88]. By trapping these CDNs, DNA-PKcs reduces their availability for STING activation and thereby dampens type I IFN responses [88]. Notably, inhibition or genetic depletion of DNA-PKcs enhanced CDN induced STING phosphorylation, IRF3 activation, inflammatory gene expression, cytokine production, and antiviral states, whereas the early response to dsDNA could still depend positively on DNA-PKcs. This dual behavior provides a mechanistic framework in which DNA-PKcs promotes the initiation of DNA-triggered immunity but later contributes to signal termination by buffering cGAMP/STING activity. Therefore, differences in stimulus type, timing of analysis, cellular background, species, DNA-PKcs abundance or catalytic status, and the relative engagement of cGAS-dependent versus cGAS-independent pathways may explain why DNA-PKcs has been reported to function as both a positive and negative regulator of innate immune signaling.

### STING-dependent and STING-independent DNA-PK signaling

The complexity of DNA-PK’s role extends to its relationship with the core cGAS–STING axis, with evidence supporting both STING-dependent and STING-independent mechanisms.

In many contexts, DNA-PKcs functions in a STING-dependent manner. For instance, DNA-PKcs can be part of a multi-protein complex including HEXIM1 and nucleoproteins (HDP-RNP), which interacts with cGAS. Upon sensing cytosolic DNA, this complex releases its components to recruit STING and activate DNA-PKcs and IRF3 [89]. Separately, the Ku70 subunit itself can act as a cytosolic DNA sensor, translocating upon DNA binding to form a complex with STING that preferentially induces the production of type III interferon (IFN-λ) [90,91].

Conversely, a STING-independent DNA sensing pathway (SIDSP) has been identified in specific human cells, such as fibroblasts and monocytes. In this pathway, DNA-PKcs can directly bind cytosolic dsDNA and, without requiring STING, recruit and phosphorylate IRF3 to drive type I IFN expression (Figure 3). This pathway is fully operational in STING-knockout cells and is abolished by the DNA-PKcs inhibitor NU7441, confirming its independence from the canonical cGAS–STING axis [92]. It should be noted that this STING-independent DNA sensing pathway has been only characterized in human fibroblasts and monocytes based on a single study [92]; its generality across other cell types and *in vivo* settings remains to be further investigated. Furthermore, the interplay between DNA-PK and cGAS-STING may also vary with the cell cycle stage, adding another layer of regulation [93].

In summary, the current landscape reveals DNA-PK as a complex and context-dependent modulator of innate immunity (Table 1). It can fine-tune the cGAS–STING pathway through direct phosphorylation or by facilitating regulatory complexes, exerting either inhibitory or stimulatory effects. Moreover, in human cells, it can orchestrate a parallel, STING-independent signaling route. These divergent findings highlight the necessity for future studies to carefully consider cell type, species, and physiological context when elucidating the intricate crosstalk between genome guardians and the immune system.

**Table 1:** Dual and context-dependent roles of DNA-PKcs in innate immunity

Functional role	Species	STING dependency	Description	Publish Year	Reference
Stimulatory	Human	Dependent	DNA-PKcs phosphorylates IRF3 Thr135 upon viral infection, stabilizes nuclear IRF3 and sustains type I IFN secretion.	2002	[87]
Stimulatory	Human and mouse	Dependent	DNA-PK complex senses cytoplasmic dsDNA via Ku, initiating STING-dependent IRF3/TBK1-mediated IFN and inflammatory cytokine production independent of DNA-PKcs kinase activity.	2012	[7]
Stimulatory	Human	Independent	DNA-PK complex senses cytosolic dsDNA and induces IRF3–IFN signaling without STING; this effect is eliminated by DNA-PKcs inhibitor NU7441.	2020	[92]
Inhibitory	Human and mouse	Dependent	DNA-PKcs phosphorylates cGAS to suppress its catalytic activity, reduce cGAMP synthesis and limit self-DNA-induced autoimmunity.	2020	[8]
Inhibitory	Human and mouse	Dependent	Viral infection triggers DNA-PKcs-dependent PARP1 cytoplasmic translocation; PARP1 PARylates cGAS to block its DNA binding and antiviral IFN response.	2022	[9]
Stimulatory	Human and mouse	Dependent	DNA-PKcs promotes IRF3 activation and IFN production in cGAS-deficient glioblastoma cells; it phosphorylates cGAS Ser435 to enhance cGAMP synthesis when cGAS exists.	2023	[11]
Stimulatory	Human	Dependent	DNA-PK complex interacts with cGAS and serves as a necessary cofactor to activate STING–IRF3 cascade after cytosolic DNA stimulation.	2024	[10]
Dual context-dependent	Human and mouse	Dependent	DNA-PKcs activates cGAS to initiate IFN response at early stages; it sequesters cGAMP to terminate sustained STING signaling in late inflammation.	2026	[88]

## DNA-PKcs in human diseases

The critical roles of DNA-PKcs in DNA repair and immune regulation are underscored by the wide spectrum of human diseases associated with its dysfunction. Aberrations in DNA-PKcs activity or expression can lead to divergent pathological outcomes, ranging from severe immunodeficiency to autoimmune disorders, neurodegenerative diseases, solid tumors, and haematological malignancies, highlighting its complex and context-dependent functions in maintaining cellular and organismal homeostasis (Figure 4).

**Figure 4:**
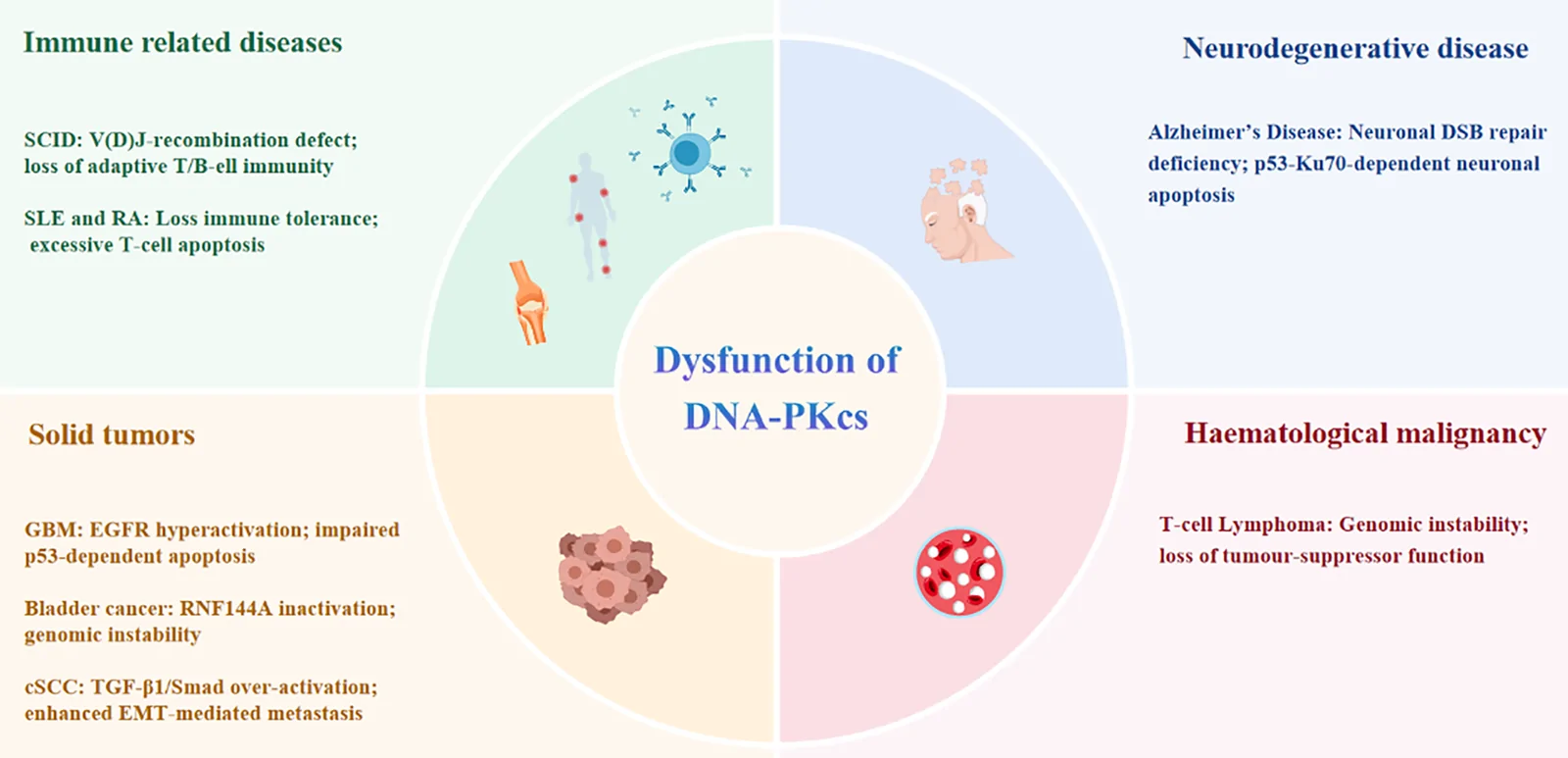
Human diseases associated with DNA-PKcs dysfunction Schematic overview illustrating major disease categories associated with dysregulated DNA-PKcs, including immunodeficiency and autoimmunity, neurodegeneration, solid tumors, and hematological malignancies. Representative disease entities and key molecular hallmarks are indicated, reflecting context-dependent pathological consequences of PRKDC genetic alterations and dysregulated DNA-PKcs activity.

### Immunodeficiency and autoimmunity: a paradoxical spectrum

The most direct link between DNA-PKcs deficiency and disease is observed in SCID. As a master regulator of the NHEJ pathway, DNA-PKcs is indispensable for the completion of V(D)J recombination during lymphocyte development. Loss-of-function mutations in the *PRKDC* gene disrupt the processing and ligation of coding ends, leading to a profound block in the generation of functional T and B cell receptors [94]. Consequently, patients present with a near-complete absence of adaptive immunity, manifesting as severe, recurrent infections early in life. The clinical phenotype can extend beyond immunodeficiency to include growth retardation, microcephaly, and neurological abnormalities, reflecting potential roles of DNA-PK in general development [95].

Paradoxically, certain *PRKDC* missense mutations that partially impair DNA-PKcs function can lead to a hyperactive innate immune state [94–96]. The mechanisms may be multifactorial, stemming from a combination of failed immune tolerance and aberrant immune activation [8,94–96].

DNA-PKcs dysfunction is implicated in SLE. Patients with SLE often exhibit high titers of autoantibodies against DNA-PKcs and the Ku complex [97]. Genetic association studies have further linked polymorphisms in NHEJ pathway genes (*XRCC5*/Ku80, *XRCC6*/Ku70, *PRKDC*/DNA-PKcs) to increased SLE susceptibility and specific clinical manifestations, such as malar rash [98]. This suggests that subtle alterations in NHEJ efficiency may contribute to a loss of immune tolerance, potentially through the accumulation of endogenous DNA damage and the subsequent breakdown of B cell receptor editing or the aberrant activation of innate immune sensors by unrepaired DNA.

In rheumatoid arthritis (RA), a different mechanism involving DNA-PKcs drives pathology. RA patient T cells display accelerated telomere shortening and accumulated DSBs [99,100]. Within this context of genotoxic stress, DNA-PKcs is hyperactivated, while Ku70/80 expression is down-regulated, creating a repair-deficit environment [101]. The persistent DSB signaling through hyperactive DNA-PKcs engages the JNK pathway, up-regulating the pro-apoptotic proteins Bim and Bmf and leading to excessive T cell apoptosis [101]. This disrupts immune homeostasis, promoting chronic immune cell turnover, immunosenescence, and sustained inflammation that fuels joint destruction. Therefore, targeting the DNA-PKcs-driven cell death pathway may represent a novel therapeutic strategy for RA.

### Neurodegeneration: DNAPK in Alzheimer’s disease

Post-mitotic neurons are particularly vulnerable to DNA damage accumulation, and a decline in DSB repair capacity is a hallmark of aging and neurodegenerative diseases [102]. In Alzheimer’s disease (AD), studies have documented a significant reduction in NHEJ activity and decreased expression of DNA-PKcs and Ku proteins in vulnerable brain regions. DNA-PKcs contributes to AD pathogenesis by directly promoting neuronal apoptosis via two interconnected pathways [103]. First, DNA-PKcs phosphorylates the tumor suppressor p53, which in turn promotes the pro-apoptotic effector Bax [104,105]. Second, DNA-PKcs phosphorylates Ku70 at Serine 6 and 51, which disrupts Ku70’s inhibitory interaction with Bax, thereby facilitating Bax mobilization and mitochondrial apoptosis [106]. This dual pro-apoptotic mechanism positions DNA-PKcs as a critical driver of neuronal loss in AD, transforming persistent DNA damage signals into an executed cell death program.

### Role of DNA-PKcs in tumorigenesis

The role of DNA-PKcs in cancer is complex and context-dependent, functioning as both a guardian of genomic integrity and, paradoxically, a facilitator of tumor progression. This functional duality underscores its potential as a therapeutic target across diverse malignancies, including solid tumors and haematological malignancies.

In certain contexts, DNA-PKcs acts as a classical tumor suppressor. As exemplified by T-cell lymphoma, a representative haematological malignancy, evidence from lymphoma models demonstrates that DNA-PKcs deficiency leads to genomic instability, accelerated aging, and a markedly increased incidence of T-cell lymphomas, highlighting its indispensable role in maintaining genomic integrity and preventing malignant transformation [107]. Conversely, in a range of solid tumors, DNA-PKcs is co-opted to drive oncogenesis. In glioblastoma (GBM), DNA-PKcs serves as a critical nexus in a non-canonical oncogenic axis [108]. This oncogenic function is likely facilitated by the molecular background of GBM, in which EGFR/receptor tyrosine kinase (RTK) activation, phosphatase and tensin homolog (PTEN) loss or inactivation, and PI3K/AKT (also termed protein kinase B, PKB) pathway activation are frequent events. TCGA-based analyses have shown that alterations involving *RTK* genes, *PI3K* genes, and *PTEN* occur in the vast majority of GBMs, while *PTEN* mutation/deletion or loss of function is particularly common in isocitrate dehydrogenase (IDH)-wildtype GBM [109–112]. These alterations may also create a permissive signaling environment for DNA-PKcs activation. Consistent with this possibility, AKT has been shown to promote post-irradiation survival of tumor cells by facilitating the initiation, progression, and termination of DNA-PKcs-dependent DSB repair [113]. In addition, evidence from melanoma models suggests that PTEN can enhance radiosensitivity by inhibiting DNA-PKcs, further supporting a functional connection between PTEN status and DNA-PKcs-mediated DNA repair, although this relationship remains to be directly validated in GBM [114]. Activated EGFR promotes the phosphorylation of DNA-PKcs, which in turn exhibits enhanced binding to the tumor suppressor p53 [108]. The formation of an EGFR/DNA-PKcs/p53 ternary complex physically impedes p53 from binding to the promoters of its target genes, such as p21 and p53 up-regulated modulator of apoptosis (PUMA) [108]. Thus, the biological outcome of DNA-PKcs depends strongly on tumor context. In lymphoma models, DNA-PKcs loss impairs DSB repair, increases genomic instability, and facilitates malignant transformation. In GBM, by contrast, DNA-PKcs acts within an oncogenic signaling background shaped by RTK/PI3K/AKT activity, PTEN deficiency in many cases, and impaired p53 tumor-suppressive output. In this setting, elevated or activated DNA-PKcs can attenuate p53-dependent cell cycle arrest and apoptosis, thereby supporting tumor progression.

Aberrant DNA-PKcs expression also plays a pivotal oncogenic role in bladder cancer and cutaneous squamous cell carcinoma (cSCC). In bladder cancer, the loss of the E3 ubiquitin ligase RNF144A disrupts the ubiquitin-mediated degradation of DNA-PKcs, leading to its abnormal accumulation [115]. This accumulation not only compromises genomic stability but also promotes tumorigenesis by driving aberrant cell differentiation, evidenced by the co-expression of basal (CK5/CK14) and luminal (GATA3) markers, and synergizing with EGFR down-regulation to fuel cancer development [115]. In cSCC, DNA-PKcs is significantly up-regulated and correlates with advanced tumor stage, metastasis, and poor prognosis [116]. It functions as a key driver of invasion and metastasis by activating the TGF-β1/Smad signaling pathway and inducing epithelial–mesenchymal transition (EMT) [116]. Inhibition or silencing of DNA-PKcs reverses EMT, suppresses cell migration and invasion *in vitro*, and significantly reduces lung metastasis *in vivo*, confirming its pro-metastatic role [116].

In summary, DNA-PKcs embodies a “double-edged sword” in oncology. Its role oscillates between a genomic guardian, as seen in lymphoma, and an active oncogenic driver in solid tumors like GBM, bladder cancer, and cSCC. This profound context-dependency, dictated by genetic background, tissue microenvironment, and upstream signaling, dictates its ultimate function in tumorigenesis and critically informs its therapeutic targeting. Table 2 summarizes DNA-PKcs-related human diseases.

**Table 2:** DNA-PKcs-related human diseases

Diseases	Description	Proposed trigger	Reference
Severe Combined Immunodeficiency (SCID)	Loss-of-function mutations disrupt NHEJ pathway, block V(D)J recombination, leading to near-complete absence of adaptive immunity.	Dysfunction in DNA-PKcs-mediated NHEJ during lymphocyte development	[94]
Systemic Lupus Erythematosus (SLE)	Polymorphisms in NHEJ pathway genes linked to increased susceptibility; patients have high titers of autoantibodies against DNA-PKcs and Ku complex.	Subtle alterations in NHEJ efficiency, accumulation of endogenous DNA damage	[97,98]
Rheumatoid Arthritis (RA)	RA patient T cells have accelerated telomere shortening and accumulated DSBs; DNA-PKcs hyperactivated, Ku70/80 down-regulated, leading to excessive T cell apoptosis via JNK pathway.	Genotoxic stress (telomere shortening, DSB accumulation)	[99–101]
Alzheimer's Disease (AD)	Significant reduction in NHEJ activity and decreased expression of DNA-PKcs and Ku proteins in vulnerable brain regions; DNA-PKcs promotes neuronal apoptosis via phosphorylating p53 and Ku70.	Persistent DNA damage signals	[103–106]
T cell lymphoma	DNA-PKcs-deficient mice show genomic instability, accelerated aging, and increased incidence of T lymphomas, indicating tumor-suppressive function in maintaining genomic integrity.	Loss of DNA-PKcs-mediated genome stability maintenance	[107]
Glioblastoma (GBM)	EGFR activation promotes DNA-PKcs phosphorylation, forming a ternary complex that blocks p53 binding to *p21*/*PUMA* promoters, inhibiting cell cycle arrest and apoptosis, and accelerating GBM progression.	EGFR activation-induced DNA-PKcs phosphorylation, leading to the formation of the EGFR-DNA-PKcs-p53 ternary complex	[108]
Bladder cancer	Abnormal accumulation of DNA-PKcs drives carcinogenesis, resulting from inactivation of E3 ubiquitin ligase RNF144A; accumulation impairs genomic stability, induces disordered cell differentiation, and synergizes with EGFR down-regulation to promote cancer.	Inactivation of RNF144A (via mutation/epigenetic silencing) leading to loss of DNA-PKcs degradation	[115]
Cutaneous Squamous Cell Carcinoma (cSCC)	DNA-PKcs activates the TGF-β1/Smad pathway to promote metastasis; its high expression is positively correlated with tumor stage, lymph node metastasis, and poor prognosis; inhibition reverses EMT and reduces lung metastases in nude mice.	Abnormal up-regulation of DNA-PKcs, leading to activation of the TGF-β1/Smad signaling pathway	[116]

## Therapeutic targeting of DNA-PKcs

The genomic instability inherent to cancer cells often coexists with a heightened dependency on DDR pathways to survive the onslaught of radiotherapy and chemotherapy. In this context, the DNA-PKcs-mediated NHEJ pathway is frequently hyperactivated, serving as a critical mechanism for maintaining genomic integrity and fostering therapy resistance. Consequently, the strategic inhibition of DNA-PKcs has emerged as a promising approach to disrupt this repair bastion, thereby sensitizing tumors to conventional DNA-damaging agents and overcoming treatment resistance.

### Inhibitor development and clinical progress

The pioneering first-generation DNA-PKcs inhibitors, such as NU7026 and NU7441, provided crucial proof-of-concept for targeting this kinase. NU7441, for instance, potently inhibits DNA-PKcs with an IC50 of 0.17–0.25 μM, effectively delaying DSB repair and significantly sensitizing cancer cells to ionizing radiation (IR) and chemotherapeutics like doxorubicin. In breast cancer models, NU7441 enhanced the cytotoxicity of IR by 4- to 12-fold and doxorubicin by 3- to 13-fold, with the most pronounced effects observed in triple-negative MDA-MB-231 cells [117]. Furthermore, in hepatocellular carcinoma (HCC), where both HR and NHEJ pathways are up-regulated, the combination of NU7441 (targeting NHEJ) with the PARP inhibitor Olaparib (targeting HR) synergistically suppressed tumor growth in both mouse models and patient-derived xenografts (PDXs) by simultaneously crippling the two major DSB repair pathways [118]. However, the clinical translation of these early compounds was hampered by suboptimal pharmacokinetic properties, including rapid systemic clearance, low oral bioavailability, and off-target effects against related kinases like PI3K and mTOR [117,119–121].

Driven by advances in medicinal chemistry and structural biology, a new generation of highly selective and drug-like DNA-PKcs inhibitors has been developed. Multiple compounds have advanced to clinical research, and relevant trial information is collated in Table 3. Peposertib (M3814) stands out as a potent and selective ATP-competitive inhibitor, boasting an IC50 of less than 3 nM for DNA-PKcs and high specificity over other PIKK family members [122]. A completed Phase I clinical trial (NCT02316197) covered patients with advanced solid tumors as well as chronic lymphocytic leukemia; the recommended Phase II dose (RP2D) was established at 400 mg twice daily (BID), with pharmacodynamic studies confirming sustained inhibition of DNA-PKcs phosphorylation in peripheral blood mononuclear cells [123]. In preclinical studies, Peposertib demonstrated robust combination efficacy. While it showed limited single-agent activity in an ovarian cancer xenograft model, the combination of Peposertib with pegylated liposomal doxorubicin (PLD) resulted in superior antitumor efficacy compared with PLD monotherapy [124]. Similarly, in HPV-positive cervical cancer models, Peposertib combined with IR led to significant and sustained tumor regression accompanied by elevated levels of γ-H2AX, underscoring its potent radiosensitizing effect [125].

**Table 3:** DNA-PKcs inhibitors and diseases

Inhibitors	Mechanism	NCT Number	Trial Phase	Cancer Type Treated	Reference
NU7441	Inhibits DNA-PKcs, delays DSB repair, and significantly enhances chemo-radiosensitivity.	–	–	Breast cancer; Hepatocellular carcinoma	[117,118]
Peposertib (M3814)	ATP-competitive inhibitor of DNA-PKcs, exhibiting potent radiosensitizing effect.	NCT02316197	Phase I	Advanced solid tumors and chronic lymphocytic leukemia	[122,123]
AZD7648	Targets PIKK regulatory domain, activates type I interferon response and promotes T cell tumor infiltration.	NCT03907969	Phase I/IIa	Advanced malignancies	[126–128]
CC-115	Simultaneously inhibits DNA-PKcs and mTOR kinase, regulating tumor growth and immune microenvironment.	NCT02833883 NCT02977780	Phase I/II	Castration Resistant Prostate and Cancer and Prostate Cancer; Glioblastoma	[132,133]
XRD-0394	Inhibits DNA-PKcs and ATM, enhances the efficacy of IR and topoisomerase I inhibitors.	NCT05002140 NCT05116254	Phase Ia/I	Locally Advanced Solid Tumor, Metastasis and Recurrent Cancer; Soft Tissue Sarcoma Adult	[134]
AsiDNA	Competitively binds to core NHEJ proteins (including DNA-binding domain of DNA-PKcs) to block repair cascade.	NCT03579628 NCT04826198	Phase I/I/II	Advanced Cancer; Ovarian Cancer	[129–131]

AZD7648 represents another structurally distinct, high-affinity inhibitor that targets the PIKK regulatory domain (PRD) of DNA-PKcs, achieving an IC50 of 0.6 nM with an excellent off-target profile [126]. This drug has entered Phase I/IIa clinical trial with trial number NCT03907969 for the treatment of advanced malignancies. In preclinical models, AZD7648 potentiated the efficacy of PLD and, notably, when combined with radiotherapy, it not only enhanced direct tumor cell killing but also activated a type I interferon response and promoted the infiltration of CD8+ T cells into the tumor microenvironment, revealing an immunostimulatory dimension to its mechanism of action [126–128].

Beyond small-molecule kinase inhibitors, an alternative strategy employs DNA mimetics. AsiDNA, a double-stranded DNA mimic molecule, competitively binds to core NHEJ proteins, including the DNA-binding domain of DNA-PKcs, thereby preventing the initiation of the repair cascade at genuine DSB sites [129]. In breast cancer models, the combination of AsiDNA and the PARP inhibitor Olaparib impaired the recruitment of repair factors like XRCC1 and RAD51/53BP1 to damage sites, leading to the accumulation of lethal DNA damage specifically in tumor cells [130]. A Phase I trial (NCT03579628) in advanced solid tumors has established a tolerable dose of 600 mg intravenous administration, supporting its further clinical development [131]. The mechanisms and clinical trial status of these and other selected DNA-PKcs inhibitors are summarized in Table 3.

### Dual-target inhibitors

The pursuit of broader pathway inhibition has led to the development of dual-target inhibitors. CC-115 concurrently inhibits both DNA-PKcs and mTOR kinase. In a Phase I trial, CC-115 demonstrated manageable toxicity at a dose of 10 mg BID, with pharmacokinetics confirming its distribution into brain tumors. It showed promising clinical activity, including a complete response lasting over four years in an endometrial cancer patient and disease stabilization in others [132]. A Phase Ib study combining CC-115 with enzalutamide in metastatic castration-resistant prostate cancer reported a recommended dose of 5 mg BID, with 80% of patients achieving a ≥50% decline in prostate-specific antigen (PSA) and a median radiographic progression-free survival of 22.1 months, particularly in patients with PI3K pathway alterations [133].

Another notable dual inhibitor, XRD-0394, targets both DNA-PKcs and ATM. This compound enhances the efficacy of IR and topoisomerase I inhibitors *in vitro* and *in vivo*. It exhibits single-agent activity in BRCA1/2-deficient cells and acts synergistically with PARP inhibitors [134]. A Phase Ia clinical trial evaluating its combination with radiotherapy (NCT05002140) has been completed, paving the way for further clinical exploration.

### Immunomodulation by DNA-PKcs inhibition

Beyond their direct role in chemosensitization and radiosensitization, DNA-PKcs inhibitors are increasingly recognized for their capacity to remodel the tumor immune microenvironment. By impeding the repair of DSBs, these inhibitors lead to the accumulation of cytosolic DNA, which acts as a potent trigger for the cGAS–STING innate immune pathway. This activation results in the production of type I interferons and pro-inflammatory chemokines, fostering an immune-permissive tumor milieu. This mechanism is robustly supported by preclinical evidence; for instance, the DNA-PKcs inhibitor NU7441 was shown to trigger a cGAS/STING-independent, IRF3-driven type-I interferon response in cGAS-deficient glioblastoma models, while also cooperating with cGAS to enhance cGAMP production and downstream signaling when both are present [11]. Furthermore, in metastatic triple-negative breast cancer models, NU7441 induced cytosolic DNA accumulation, activated the cGAS–STING and retinoic acid-inducible gene I (RIG-I) pathways, and fostered an inflamed tumor microenvironment characterized by increased infiltration of CD8+ T cells and CD11c+ dendritic cells, ultimately promoting anti-tumoral immunity in an immune-competent host [135]. These findings underscore a dual antitumor mechanism for DNA-PKcs inhibitors: they directly compromise genomic integrity through repair inhibition while simultaneously activating a potent, innate immune-derived antitumor response. Table 3 summarizes DNA-PKcs inhibitors and their therapeutic profiles.

### Barriers limiting clinical translation of DNA-PKcs inhibitors

Various DNA-PKcs inhibitors exhibit potent anti-tumor activity in a wide range of preclinical tumor models; nevertheless, numerous candidate agents yield unsatisfactory clinical outcomes in human studies or face premature trial termination. These obstacles can be attributed to three major categories: inherent molecular defects of drug candidates, dose-limiting toxicities toward normal tissues, and therapeutic resistance developed by tumors via compensatory DNA repair pathways.

First, the majority of DNA-PK-targeted agents suffer from intrinsic molecular flaws, including suboptimal pharmacokinetic profiles and insufficient kinase selectivity. Frequently encountered drawbacks consist of poor aqueous solubility, unstable *in vivo* metabolism and short serum half-lives. The first-generation lead compound NU7441 undergoes rapid systemic clearance and possesses extremely low oral bioavailability, making it difficult to maintain therapeutically effective concentrations within solid tumors and achieve sustained intratumoral suppression of DNA-PKcs [120]. Even AZD7648, a second-generation selective inhibitor, demonstrates limited tumor penetration at clinically tolerable doses and fails to achieve sufficient target engagement. Most ATP-competitive DNA-PK inhibitors also exhibit inadequate selectivity. Early NU-series compounds non-specifically suppress other PIKK family proteins such as ATM, Ataxia telangiectasia and Rad3-related protein (ATR), mTOR, and PI3K, disrupting multiple physiological signaling cascades and exacerbating adverse reactions [119,126]. As a dual mTOR/DNA-PK inhibitor, CC-115 encounters an additional challenge at its clinically recommended dose: although the drug effectively blocks mTOR signaling, it cannot sufficiently inhibit DNA-PKcs, which substantially impairs the anticipated synergistic effects of DNA repair suppression and radiosensitization [133]. Distinct from small-molecule kinase inhibitors, the oligonucleotide decoy AsiDNA has unique inherent limitations. Devoid of intrinsic DNA-damaging activity, AsiDNA shows no cytotoxicity as monotherapy and must be combined with DNA-damaging treatments to exert anti-tumor effects. Moreover, its synergistic efficacy is highly dependent on the PARP inhibitory potency of combination partners; combination with weak PARP inhibitors such as iniparib only produces marginal sensitization, greatly restricting the pool of viable combinatorial regimens [130]. Accordingly, developing novel compounds with improved physicochemical properties and enhanced kinase selectivity is critical to break the clinical translational bottleneck of this class of agents.

Second, DNA-PKcs fulfills essential physiological functions in repairing DNA double-strand breaks in normal cells, resulting in a narrow therapeutic window for these inhibitors. When combined with radiotherapy or DNA-damaging chemotherapy, DNA-PKcs inhibitors concurrently damage rapidly proliferating normal tissues including bone marrow and gastrointestinal epithelium, triggering dose-limiting toxicities such as severe anemia, gastrointestinal injury and mucosal inflammation [120]. The Phase I/II clinical trial of AZD7648 combined with pegylated liposomal doxorubicin was terminated early due to unmanageable cumulative toxicities. Only a small subset of patients achieved partial tumor regression, and broad therapeutic benefits were not observed [126]. Clinical investigation of CC-115 combined with enzalutamide for metastatic castration-resistant prostate cancer further confirms such safety risks [133]. Simultaneous inhibition of mTOR and DNA-PK pathways tends to induce severe cutaneous adverse events, predominantly grade 3 rash and persistent pruritus. These cutaneous toxicities represent one of the key reasons for mandatory dose reduction, restricting the recommended Phase 2 dose to 5 mg twice daily and preventing further dose escalation.

Third, tumors activate alternative compensatory repair pathways to develop therapeutic resistance and attenuate inhibitor efficacy. Tumor cells can initiate the HR pathway to compensate for impaired NHEJ resulting from DNA-PKcs suppression, counteract drug-induced DNA damage, and ultimately acquire therapeutic resistance [136]. Long-term administration of CC-115 drives transcriptional up-regulation of the HR pathway in tumor cells, gradually inducing acquired resistance and reducing the durable anti-tumor activity of the drug [133]. Among available candidate agents, M3814 displays limited anti-tumor activity as monotherapy. After advancing into clinical trials, most DNA-PKcs inhibitors can only induce tumor shrinkage in a minority of patients and fail to achieve universal therapeutic responses [122].

In summary, unfavorable pharmacokinetic characteristics, insufficient kinase selectivity, inadequate target suppression of dual-target agents, unique mechanistic constraints of the oligonucleotide AsiDNA, unavoidable dose-limiting injury to normal tissues, and tumor resistance mediated by compensatory DNA repair mechanisms collectively constitute the core barriers hindering the clinical translation of DNA-PKcs inhibitors and contributing to the premature termination of multiple clinical trials.

## Conclusion and future perspectives

Over the past decades, research has unequivocally established DNA-PKcs as a quintessential multifunctional molecule, occupying a central node at the critical intersection of DSB repair, adaptive immunity, and innate immune signaling. Its dysfunction is intimately linked to a spectrum of human diseases, ranging from severe immunodeficiency and autoimmunity to cancer and neurodegeneration, underscoring its fundamental role in maintaining cellular and organismal homeostasis.

Our understanding of DNA-PKcs in its canonical role has matured significantly. The architectural blueprint of the DNA-PK holoenzyme, the functional significance of key phosphorylation clusters (e.g., ABCDE, PQR, and JK) in orchestrating the assembly and disassembly of the NHEJ machinery, and its indispensable function in processing coding ends during V(D)J recombination are now well-defined. These insights provide a solid mechanistic foundation for understanding diseases like SCID. In contrast, the role of DNA-PKcs in the cytoplasm, particularly in cytosolic DNA sensing and the regulation of the cGAS–STING pathway, presents a more complex and still unfolding narrative. Compelling evidence positions DNA-PKcs as both an inhibitor and an activator of this pathway, with its function likely determined by cellular context, the nature of the stimulus, post-translational modifications, and notable species-specific differences. Key questions remain largely unanswered: How do the expression levels and PTM status of DNA-PKcs in different cell types dictate its immunomodulatory output? What are the precise components of the STING-independent DNA–PKcs–IRF3 axis, and how does it crosstalk with the canonical cGAS–STING pathway?

In disease pathogenesis, while the link between complete loss-of-function mutations and SCID is clear, the consequences of more subtle dysregulation are less so. The contribution of polymorphisms in NHEJ genes (e.g., *XRCC5*, *XRCC6*) to polygenic autoimmune diseases like SLE, often in concert with environmental triggers like oxidative stress, requires validation in large-scale clinical cohorts and deeper mechanistic exploration. Similarly, in Alzheimer’s disease, the detailed molecular interplay between DNA-PKcs, the Ku complex, and the pro-apoptotic machinery (p53/Bax, Ku70/Bax) in driving neuronal loss demands further elucidation.

Looking forward, we envision progress in three key areas to propel the DNA-PK field forward and translate basic knowledge into clinical benefit: (1) Mechanistic Decoding at High Resolution. Future research must leverage cutting-edge technologies to capture the dynamic nature of DNA-PKcs. Cryo-electron microscopy (cryo-EM) can visualize the conformational landscapes of DNA-PKcs in different functional states, such as DNA-bound, kinase-active, or engaged with immune sensors. Advanced proteomics and single-cell multi-omics will be crucial to map the context-dependent interactome and PTM landscape of DNA-PKcs, revealing how these modifications serve as a molecular switch between its repair and immune functions. A major goal is to define the precise structural interfaces governing its interactions with cGAS, IRF3, and other innate immune components. (2) Bridging Basic Biology and Clinical Translation. The development of DNA-PKcs inhibitors has opened a new therapeutic frontier, with the current focus on optimizing their drug-like properties, such as oral bioavailability and off-target effects, through rational drug design. In parallel, well-designed multicenter clinical trials are imperative to explore innovative combination strategies. These include pairing DNA-PKcs inhibitors with immune checkpoint blockers to leverage their immunostimulatory potential, or with PARP inhibitors in HR-deficient cancers to induce synthetic lethality. The success of these strategies will hinge on the development of robust companion diagnostics, potentially based on DNA-PKcs expression or functional activity, to identify patient populations most likely to benefit. (3) Expanding the Therapeutic Horizon. Beyond oncology, the role of DNA-PKcs in autoimmune and neurodegenerative diseases suggests a broader therapeutic potential. Modulating DNA-PKcs activity represents a novel but challenging avenue: this involves either its inhibition in contexts of hyperactive pro-apoptotic signaling (e.g., RA and AD) or its careful potentiation in immunodeficiency states. Furthermore, understanding how DNA-PKcs integrates metabolic and genotoxic stress signals could reveal its role in aging and other physiological processes.

In conclusion, DNA-PKcs has emerged from being a dedicated nuclear “DNA repair specialist” to a versatile “molecular integrator” at the nexus of genome integrity and immunity. The journey ahead is to deconvolute its complex biology with higher precision, harness its dual nature for therapeutic gain across a spectrum of diseases, and ultimately realize its full potential as a cornerstone of next-generation precision medicine.

## Data Availability

No data were used for the research described in the article.

## Abbreviations

AD: Alzheimer’s disease
AKT: protein kinase B (PKB)
ATM: ataxia-telangiectasia mutated
ATR: ataxia telangiectasia and Rad3-related protein
BID: twice daily
c-NHEJ: canonical non-homologous end joining
cGAS-STING: cyclic GMP–AMP synthase and stimulator of interferon genes
CRL4A^DTL^: Cullin-RING ubiquitin Ligase 4A complex with substrate receptor DTL
DAMP: damage-associated molecular pattern
DDR: DNA damage response
DNA-PKcs: DNA-dependent protein kinase catalytic subunit
EGFR: epidermal growth factor receptor
EMT: epithelial–mesenchymal transition
FAT: FRAP–ATM–TRRAP
FATC: FAT C-terminal motif
FRB: FKBP12–rapamycin-binding
GBM: glioblastoma
HCC: hepatocellular carcinoma
HEAT: Huntingtin, Elongation factor 3, Protein phosphatase 2A, and TOR1
HR: homologous recombination
HSV-1: Herpes Simplex Virus-1
HUWE1: HECT, UBA, and WWE domain containing E3 ubiquitin protein ligase 1
IDH: isocitrate dehydrogenase
IFN-λ: Type III interferon
IFN: Type I interferon
IR: ionizing radiation
IRF3: interferon regulatory factor 3
mTOR: mechanistic target of rapamycin kinase
MVA: Modified Vaccinia Ankara
NHEJ: non-homologous end joining
PARP1: poly(ADP-ribose) polymerase 1
PDX: patient-derived xenograft
PI3K: phosphatidylinositol 3-kinase
PIKK: phosphatidylinositol 3-kinase-related kinase family
PLD: PEGylated liposomal doxorubicin
PP1: protein phosphatase 1
PRD: PIKK regulatory domain
PRR: pattern recognition receptor
PSA: prostate-specific antigen
PTEN: phosphatase and tensin homolog
PTM: post-translational modification
PUMA: p53 up-regulated modulator of apoptosis
RA: rheumatoid arthritis
RAG1/RAG2 complex: recombination-activating gene 1/recombination-activating gene 2 complex
RIG-I: retinoic acid-inducible gene I
RNF144A: Ring Finger protein 144A
RP2D: Recommended Phase II dose
RTK: receptor tyrosine kinase
SCID: severe combined immunodeficiency
SIDSP: STING-independent DNA sensing pathway
SIRT2: Sirtuin 2
SLE: systemic lupus erythematosus
SMYD2: SET and MYND domain-containing protein 2
TBK1: TANK-binding kinase 1
USP7: ubiquitin-specific protease 7
WASH: Wiskott–Aldrich Syndrome Protein and SCAR Homolog
